# An exploration of biosimilar TNF-alpha inhibitors uptake determinants in hospital environments in Italy, Portugal, and Spain

**DOI:** 10.3389/fmed.2022.1029040

**Published:** 2023-01-10

**Authors:** Teresa Barcina Lacosta, Arnold G. Vulto, Isabelle Huys, Steven Simoens

**Affiliations:** ^1^Department of Pharmaceutical and Pharmacological Sciences, KU Leuven, Leuven, Belgium; ^2^Hospital Pharmacy, Erasmus University Medical Center, Rotterdam, Netherlands

**Keywords:** biosimilars, off-patent biologics, Italy, Portugal, Spain, TNF-alpha, policies, uptake

## Abstract

**Background:**

The availability of biosimilar medicines in Southern European markets has allowed purchasing biologics at a lower cost for healthcare systems. However, the capacity to seize this cost-reduction opportunity in the long run depends on fostering a sustainable competitive environment for all the market players involved. Diverse policies and information campaigns have been launched in Italy, Portugal and Spain to support uptake of “best-value” biologics (BVB). Despite these measures, the utilization of lower-cost biologics in certain regions is low, especially when it comes to the treatment of chronic conditions.

**Objective:**

We aim to identify biosimilar uptake determinants in hospital environments in Italy, Portugal and Spain, using the class of TNF-alpha inhibitors as an example.

**Methods:**

This is a mixed-methods study based on (1) the quantitative analysis of regional uptake data for TNF-alpha inhibitor biosimilars and (2) the qualitative processing of semi-structured interviews capturing experts’ views on uptake determinants for biosimilars.

**Results:**

The organization of multi-stakeholder information campaigns supporting TNF-alpha inhibitor biosimilars use in Italy, Portugal and Spain has resulted in an increased familiarity of healthcare professionals and patients with the prescription/use of these products. However, barriers persist that impede high biosimilars uptake, especially in chronic patient populations eligible for a switch. These are: (1) the late publication of position statements on biosimilars interchangeability by regulatory health authorities; (2) the vague positioning of (national/regional) health authorities on best switching practices (including multiple biosimilar-to-biosimilar switches); (3) the existence of policy frameworks that do not necessarily support the initiation of switching protocols; (4) the establishment of sometimes inefficient purchasing procedures that limit biosimilars potential to compete for market shares. Diverse approaches taken regionally to address these barriers have contributed to heterogeneous TNF-alpha inhibitor biosimilars uptake across Southern Europe.

**Conclusion:**

Our research signaled the limited reach of biosimilar policies implemented locally, if not supported by a national policy framework. This study highlights the need for the coordinated implementation of policy measures fostering biosimilars use at the regional and national level in Italy, Portugal and Spain. These measures should account for the particularities of off-patent biologic and biosimilar markets and should jointly address supply- and demand-side challenges.

## 1. Introduction

Fifteen years of biosimilars availability in Europe have shown that biosimilar markets do not operate like generic medicines markets. The structural and manufacturing complexity of biologics has led to higher market-entry costs for these products than for generics. Besides, the literature indicates that: (1) not every off-patent biologic is exposed to biosimilars competition; (2) biosimilars market access is often delayed; (3) biosimilars availability does not always lead to strong price competition (1, 2). In the clinical practice, the fact that EMA-approved biosimilars are considered therapeutically equivalent to the reference product has not necessarily translated into providers and patients preferentially choosing “best-value” biologics (i.e., lower-priced originator biologics or biosimilars) over more costly biologics. Therefore, the demand for biologics is not always price sensitive, as indicated by Moorkens et al. in a study investigating uptake drivers for etanercept biosimilars in Sweden (3).

Implemented pharmaceutical policies, as well as countries’ political and socio-economic environments, are known to affect biosimilars uptake. The coordination of policies for biosimilars market entry, pricing, purchasing, reimbursement, and uptake at the individual Member States level, explains observed country-specific biosimilar adoption patterns. Further, it is known that the adoption of biosimilars in a country varies with the care setting, the therapeutic area and the drug class (4–6). Diverse European experiences have shown the advantages of complementing biosimilar policies targeting biologic-naïve patients with policies aimed at biologic-experienced populations (e.g., switching policies). This can be critical to ensure sufficient biosimilars sales volume in therapies indicated for chronic conditions (e.g., TNF-alpha inhibitor therapies). In Europe, the individual Member States are responsible for regulating biosimilars interchangeability (i.e., the possibility of exchanging one medicine for another therapeutically equivalent medicine), switching (i.e., a prescriber-initiated practice to exchange one medicine for another with the same therapeutic intent) and automatic substitution (i.e., a pharmacy-led practice to exchange one medicine for another with the same therapeutic intent). Coordination of switching policies at the Member State level has resulted in variable approaches; from the full endorsement of switching practices and the establishment of top-down mandated switching protocols to more cautious approaches, and even in some cases, to the lack of official position statements on interchangeability and switching (7, 8). A cautious approach toward the interchangeable use of biologics and the establishment of switching protocols has been adopted by institutions in Southern Europe (i.e., Italy, Portugal and Spain). Only since 2018, the Italian Medicines Agency (AIFA) considers that EMA-approved biosimilars are interchangeable with the corresponding originator and endorses the switch of biologic-experienced patients (9). The Medicines Agencies in Portugal and Spain have also acknowledged the quality of EMA-approved biosimilars but consider that interchangeability should be established at the level of Pharmacy and Therapeutics Committees within hospitals (10–12). This has led to diverse switching practices implemented at the regional and hospital level.

To account for the country-, setting-, and product-specific nature of biosimilar uptake patterns, the current study aims to investigate uptake determinants for TNF-alpha inhibitor biosimilars at the hospital level in Italy, Portugal, and Spain. TNF-alpha inhibitors have an important role in the treatment of chronic systemic immune-mediated conditions such as rheumatoid arthritis, ankylosing spondylitis, psoriatic arthritis and inflammatory bowel diseases. Five TNF-alpha inhibitor molecules have received marketing approval by the EMA: infliximab, etanercept, adalimumab, certolizumab pegol, and golimumab. Biosimilars for infliximab, etanercept, and adalimumab have been available in these Southern European markets since 2015, 2016, and 2018, respectively. It is hypothesized that variability in the implementation of switching and other demand-side policies, together with variability in procurement conditions for biologics, has led to high intra-country heterogeneity in the uptake of TNF-alpha inhibitor biosimilars in Southern Europe.

## 2. Materials and methods

This study is a mixed methods study based on: (1) a literature review, (2) the quantitative analysis of biosimilar market shares for hospital-use TNF-alpha inhibitors in Italy, Portugal, and Spain, and (3) the qualitative analysis of semi-structured interviews gathering experts’ views on biosimilar uptake determinants. Market data provided on TNF-alpha inhibitor biosimilars uptake were analyzed at the national and regional level. Findings from the market data analysis were discussed in semi-structured interviews with experts across Italy, Portugal and Spain. The general narrative literature review conducted was aimed at complementing findings from the quantitative analysis and the conducted interviews. Due to the complementary nature of the methods used, the study’s approach has allowed addressing limitations in data availability. Scarce information has been published in the literature interpreting biosimilar uptake data at the national/regional level in Italy, Portugal, and Spain. Also, little is known about implemented biosimilar policies at the regional level in these countries. In this sense, our study relies on the expertise of interviewees. It is relevant to note that there is an overlap with results from the literature review and the interviews. In multiple occasions, the interviewer referred to published documents in order to get clarifications from interviewees on the content. Also, interviewees pointed to additional websites and documents that served to inform our study, and to provide more detailed information on the topics discussed. Based on the complementarity of the research methods used, the learnings from this study are reported in an integrated way, with references to the literature to provide context for the quantitative and the qualitative part.

### 2.1. Literature review

We conducted a general narrative literature review to describe the main characteristics of the Portuguese, Italian, and Spanish markets for TNF-alpha inhibitors, and to identify biosimilar policies implemented in these countries. The search strategy was based on the screening of scientific databases (Google scholar, PubMed/Medline, Embase) and gray literature between October 2021 and April 2021. Academic databases were searched to yield information on combined searches including the terms: “supply-side,” “demand-side,” “policies,” “measures,” “procurement,” “tendering,” “framework agreements,” “quotas,” “prescription targets/objectives,” “market-shares,” “uptake,” “TNF-alpha inhibitors,” “infliximab,” “etanercept,” “adalimumab,” “golimumab,” “certolizumab pegol,” “biosimilars,” “biologics,” “originator biologics,” “reference biologics” and, “Italy,” “Portugal,” and “Spain.” These terms were adapted to the nomenclature of each specific database. Apart from screening academic databases, and in order to collect country-specific information on biosimilar policies, we consulted gray literature repositories within websites of Italian, Portuguese and Spanish health institutions (e.g., AIFA, OsMed, Infarmed, and AEMPS) and related organizations (e.g., ACSS, SPMS, BioSim, INGESA, and AIReF; see glossary of terms in Table 1). We included full-text publications, conference abstracts, posters and institutional document published in the time period 2010–2022. We selected documents written in English, Italian, Portuguese, and Spanish.

**TABLE 1:** Glossary of specialized abbreviations and terms used in the article.

Term	Meaning in English	Meaning in the native language
ACSS	Central Administration of the Health System, Portugal	Administração Central do Sistema de Saúde
AEMPS	Spanish Medicines Agency	Agencia Española de Medicamentos y Productos Sanitarios
AIFA	Italian Medicines Agency	Agenzia Italiana del Farmaco
AIReF	Independent Authority for Fiscal Responsibility, Spain	Autoridad Independiente de Responsabilidad Fiscal
ARS	Regional Health Administration	Administração Regional de Saúde
BioSim	The Spanish Association of Biosimilar Medicines	Asociación Española de Medicamentos Biosimilares
CFT	Pharmacy and Therapeutics Committee (hospital), Portugal	Comissão de Farmácia e Terapêutica
CIPM	Interministerial Pricing Commission	Comisión Interministerial de Precios de Medicamentos y Productos Sanitarios
CNFT	National Pharmacy and Therapeutics Committee, Portugal	Comissão Nacional de Farmácia e Terapêutica
CURMP	Technical Committee for the Rational Use of Medicines, Spain (Asturias)	Comisión de Uso Racional de Medicamentos y Productos Sanitarios
EMA	European Medicines Agency	–
Infarmed	Portuguese National Authority of Medicines and Health Products	Autoridade Nacional do Medicamento e Produtos de Saúde
INGESA	National Institute of Healthcare Management, Spain	Instituto Nacional de Gestión Sanitaria
OsMed	AIFA’s Medicines Utilization Monitoring Centre	Observatorio Nazionale sull’Impiego dei Medicinali
RHAs	Regional Health Authorities	–
SEFH	Spanish Society of Hospital Pharmacy	Sociedad Española de Farmacia Hospitalaria
SNS	Portuguese National Health Service	Serviço Nacional de Saúde
SNS	Spanish National Health Service	Sistema Nacional de Salud
SPMS	Shared Services of Ministry of Health, Portugal	Serviços Partilhados do Ministério da Saúde
SSN	Italian National Health Service	Servizio Sanitario Nazionale
TACRC	Central Administrative Court on Contracts, Spain	Tribunal Administrativo Central de Recursos Contractuales
TAR	Regional Administrative Courts, Italy	Tribunale Amministrativo Regionale

English terms and the equivalent Italian, Portuguese, and Spanish terms are included.

### 2.2. Analysis of TNF-alpha inhibitor biosimilars market shares

**FIGURE 1:**
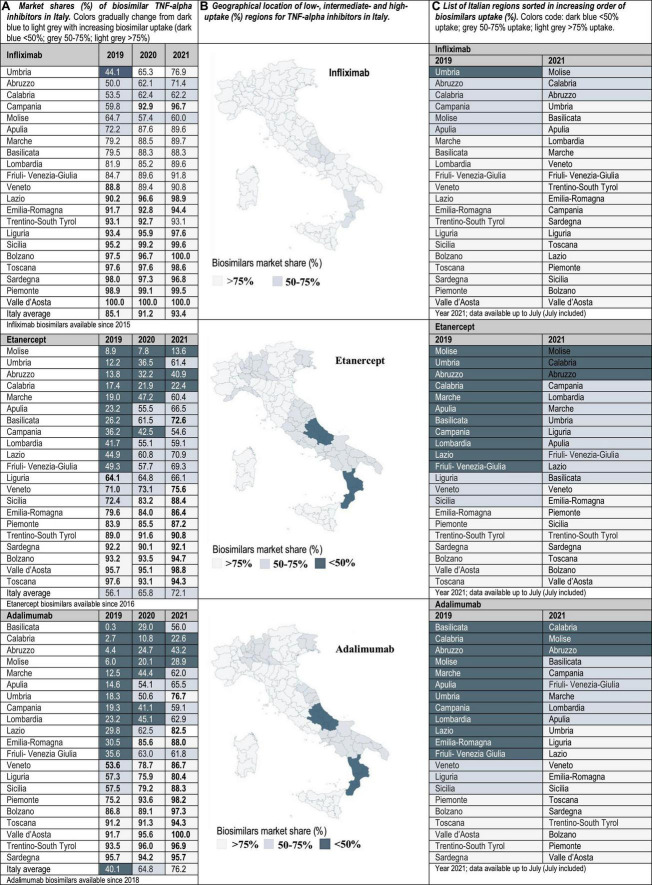
**(A)** Market shares (%) of biosimilar TNF-alpha inhibitors in Italy. Data at the national/regional level are provided for infliximab, etanercept, and adalimumab biosimilars (2019–2021). Regional uptake data were only publicly available since 2019. **(B)** Geographical location of low-(<50%), intermediate-(50–75%), and high-uptake (>75%) regions for TNF-alpha inhibitors in Italy. **(C)** List of Italian regions sorted in increasing order of biosimilars uptake (%).

**FIGURE 2:**
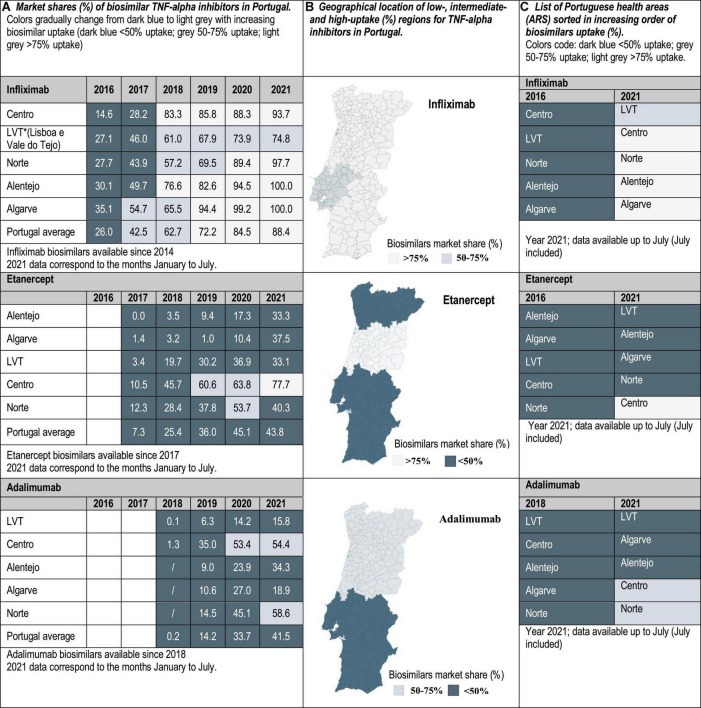
**(A)** Market shares (%) of biosimilar TNF-alpha inhibitors in Portugal. Data at the national/regional level are provided for infliximab (2016–2021), etanercept (2017–2021), and adalimumab biosimilars (2018–2021). **(B)** Geographical location of low-(<50%), intermediate-(50–75%), and high-uptake (>75%) regions for TNF-alpha inhibitors in Portugal. **(C)** List of Portuguese health areas sorted in increasing order of biosimilars uptake (%).

**FIGURE 3:**
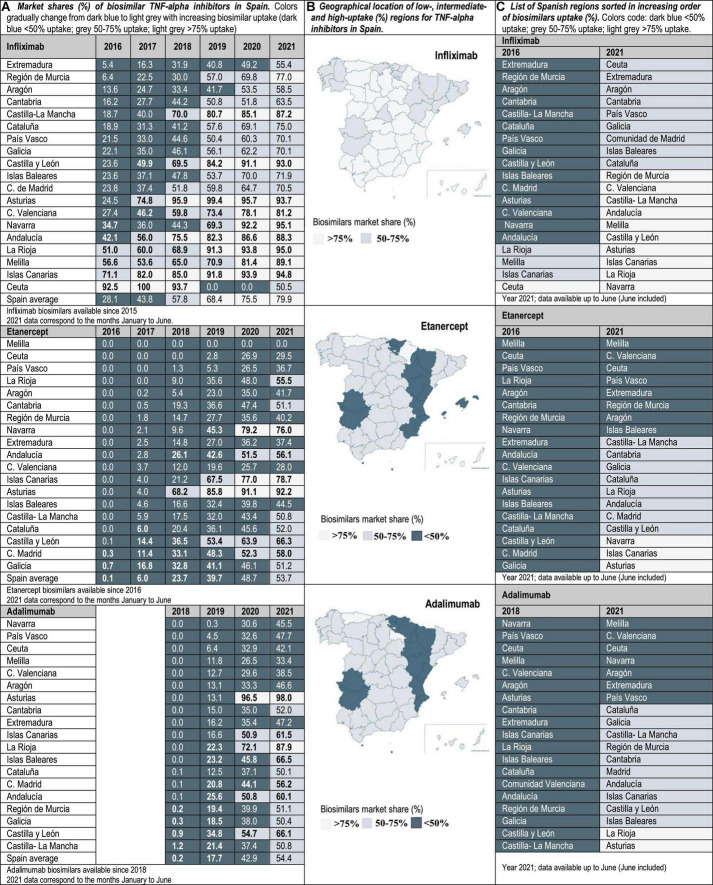
**(A)** Market shares (%) of biosimilar TNF-alpha inhibitors in Spain. Data at the national/regional level are provided for infliximab (2016–2021), etanercept (2016–2021), and adalimumab biosimilars (2018–2021). **(B)** Geographical location of low-(<50%), intermediate-(50–75%), and high-uptake (>75%) regions for TNF-alpha inhibitors in Spain. **(C)** List of Spanish regions sorted in increasing order of biosimilars uptake (%).

Drug utilization data for TNF-alpha inhibitor biosimilars, reported as biosimilar market shares (%), were examined from 2016 to 2021 in the case of Portugal and Spain, and from 2019 to 2021 in Italy. Aggregated market shares for infliximab, etanercept and adalimumab biosimilars were expressed as the sales volume of all marketed biosimilar products over the volume of biosimilars plus the volume of the respective originator product. The study time frame was determined by data availability. The analyzed data were provided by the AIFA Medicines Utilization Centre (OSMED) in Italy (13, 14), by the National Authority of Medicines and Health Products (Infarmed) in Portugal (15), and by the Spanish Association of Biosimilar Medicines (BioSim) and the Ministry of Health Department of Pharmaceuticals and Health Products in Spain (16, 17). The analysis of biosimilars uptake at the regional level allowed the identification of high-and low-uptake regions. As indicated in Figures 1–3, biosimilar market shares above 75% were considered to be high and biosimilar market shares below 50% were considered to be low (see data classified according to a color code in Figures 1–3).

### 2.3. Qualitative analysis of semi-structured interviews

Insights from Italian, Portuguese and Spanish experts regarding determinants of TNF-alpha inhibitor biosimilars uptake were gathered via semi-structured interviews. A total of 10 interviews were conducted from November 2022 to March 2022. In Table 2 we provide a summary of the participants’ background. Considering the specific area of expertise required for participation in this study, and in order to not only ensure the confidentiality of participating individuals but also of participating institutions, we only provide information on how many participants were enrolled per country (see Table 2). The purpose of conducting these interviews was to describe supply- and demand-side considerations that may have affected biosimilars adoption in clinical practice. Purposive sampling was applied to ensure the inclusion of participants with different backgrounds (i.e., regulatory, industry, academia, clinical), and to be able to describe supply- and demand-side policies affecting biosimilars use. The interviews were conducted in English when involving Italian and Portuguese stakeholders, and in Spanish when involving stakeholders from Spain. Potential participants were contacted via e-mail and the interviews were conducted online through Microsoft Teams or Skype for Business (45–60 min duration). Interview participants were informed in advance about the aims of the research project, the type of funding received to support this project, and the main interview topics. Although participants did not have access to the full interview guide in advance, they received an informed consent form explaining the scope of the study and the conditions for data processing (in accordance with the GDPR Belgium Law). If agreed by participants, the interviews were audio-recorded and transcribed *verbatim*. When the audio recording was not possible, notes were taken to write a summary report with the main conversation highlights. The shared informed consent form anticipated the possibility to conduct follow-up interviews with each participant. As the participants addressed all the questions formulated by the interviewer in the first round of interviews, it was not considered necessary to proceed with follow-up interviews. However, participants were given the possibility to follow-up with the researcher for more information about data analysis and reporting.

**TABLE 2:** List of interviews conducted and summary of the participants’ background.

Interviewees’ background	Number of interviews conducted
Academia—Expertise in patent law and competition	1
Academia—Expertise in medicines procurement	1
Academia—Expertise in pharmaceutical policies	1
Hospital pharmacist	4
Industry representative	1
Regulator—Expertise in pharmaceutical policies	1
Regulator/health institution representative— Expertise in medicines procurement	1

A total of 10 interviews were conducted with expert stakeholders in Italy, Portugal, and Spain. Other health policy and procurement experts with an academic affiliation (not indicated in this table) contributed to our research by providing written information on market data for TNF-alpha inhibitors in Southern Europe, and by informally discussing the implications of implemented biosimilar policies. Number of interviews conducted per country: Italy (2), Portugal (4), Spain (4).

We utilized an interview guide (26 open-ended questions) that was approved (7 September 2021) by the UZ/KU Leuven ethics committee (reference number: S65745). The selection of interview questions was based on the structure of a guide previously developed by our research group to study market and uptake dynamics for TNF-alpha inhibitors in Germany and Sweden (3–5). The interview guide topics were adapted to specific characteristics of Italian, Portuguese and Spanish TNF-alpha inhibitor markets. Interview questions were classified based on the themes: supply-side considerations (sub-themes: pricing, procurement, and reimbursement procedures for biologics) and demand-side considerations (sub-themes: biosimilars uptake and initiatives established to foster biosimilars use) (see interview guide within the Supplementary material). All interviews were carried out by the same researcher, having a background in pharmacy and over 3 years of experience with conducting market data analyses for biologics and with applying semi-structured interview techniques. As a relationship was not established with participants prior to study commencement, during the introductions section of the interview, information was given about the background and expertise of the interviewer. The transcripts and the interview notes were pseudonymized and processed via manual coding using the software QSS NVivo 12. The same researcher that conducted the interviews transcribed the content, developed the strategy for data analysis and coded the data. A thematic analysis approach was chosen. This required the transcription of interview recordings and the coding of the content of the transcripts. Initially, the researcher read the interview transcripts repeatedly to become familiar with the content, and to evaluate whether pre-defined interview themes were addressed, and whether additional themes emerged. It was possible to apply open coding for the more inductive aspects of the study. The second level of analysis involved the categorization of identified sub-themes (i.e., the identification of interrelated sub-themes and the grouping of these sub-themes into higher level themes). It was decided to organize the reporting of the results for each country according to the main interview themes: supply- and demand-side considerations. Data provided within the results section of the manuscript were critically reviewed and approved for publication by all the authors.

## 3. Results

Interviewed experts indicated that the level of familiarity of healthcare professionals (HCPs) and patients with the prescription/use of biosimilars has increased in the immunology area. This observation is supported by data showing that the latest market additions (i.e., adalimumab biosimilars) have had the highest uptake growth rate. Interviewees have generally attributed the increased familiarity with biosimilars in Italy, Portugal and Spain to the organization of multi-stakeholder education campaigns on the efficacy and safety of using biosimilars. However, despite more than 5 years of experience with the interchangeable use of TNF-alpha inhibitor biosimilars in the clinical practice, interviewed hospital pharmacists still expressed doubts concerning how to best introduce these medicines in populations of biologic-experienced patients that receive chronic treatment and are stable on an originator product. This is a special concern for HCPs in the case of self-administered subcutaneous formulations, considering that patients are aware of changes in administration devices introduced because of a switch. According to interviewed hospital pharmacists, it is sometimes unclear whether cost-effectiveness criteria should dictate a switch, and how to regulate multiple biosimilar-to-biosimilar switches. Differences in criteria have led to areas where eligible patients are routinely switched to BVB in all relevant clinical departments, and to areas where clinical departments are resistant to initiate a switch. According to our analysis of market data, these differences in criteria are reflected in the heterogeneous TNF-alpha inhibitor biosimilars uptake levels. In the three countries of study, the lowest uptake variability range (25, 40, and 45%) was observed for infliximab, and the highest (45, 85, and 92%) for etanercept in Portugal, Italy and Spain, respectively (see Figures 1–3). In the following sections we interpret TNF-alpha inhibitor biosimilars uptake data in the light of supply- and demand-side policies implemented to regulate biosimilars use in Italy, Portugal, and Spain. Table 3 lists factors that have influenced biosimilars uptake in the three countries of study.

**TABLE 3:** Identified factors influencing TNF-alpha inhibitor biosimilars uptake in Italy, Portugal, and Spain.

Qualitative analysis—Factors influencing low biosimilars uptake within the TNF-alpha inhibitors class
**>Molecule- and product-related factors** **Administration route:** subcutaneous administration/self-injection device **Approved indications:** chronic conditions Uncertainties persist on how to best introduce biosimilars in populations of biologic-experienced patients that receive chronic treatment and are stable on the originator product. This is a special concern in the case of self-administered subcutaneous formulations, considering that patients are aware of changes in administration devices

**>Evolution in the standards of care; shifts in utilization patterns from off-patent TNF-alpha inhibitors toward on-patent immunomodulators**

**>Procurement/purchasing procedures that limit biosimilars potential to generate market competition** E.g., time inefficient establishment of centralized procedures, reliance on negotiations parallel to public/centralized purchasing procedures to cover for demands of biologics not awarded a supply contract, parallel negotiations giving exclusivity to originator manufacturers for biologic-experienced populations, procedures that put excessive pressure on prices and supply (single-winner/single-award criteria)

**>Health authorities’ late/vague positioning regarding biologics interchangeability and best switching practices** (in relation to other European countries) (8)

**>Reliance on legal frameworks that do not necessarily support the initiation of switching protocols**

**>Unequal distribution of healthcare systems’ resources**

**>Limited resources for benchmarking and coordination** This affects the capacity to coordinate criteria for best switching practices at the regional and national level, and to coordinate prescription criteria between private and public institutions

### 3.1. The TNF-alpha inhibitors market in Italy

In Italy, the planning of health services is organized by the Regional Health Authorities (RHAs) that cover the 21 administrative jurisdictions. However, it is the Italian National Health Service (SSN) who retains the overall responsibility over the health budget and sets the ceiling for regional hospital pharmacy spending. In parallel, the Italian Medicines Agency (AIFA) oversees pricing and reimbursement policies for medicines. According to these policies, biosimilars are automatically placed in the same reimbursement class as originator biologics, if their price is set at least 20% lower than the originator’s price (18, 19).

It is the regions’ competence to purchase medicines and to control pharmaceutical spending. When the expenditure ceiling is surpassed, half of the excess is charged to the regional government, and the other half to the pharmaceutical industry. This has encouraged the implementation of measures controlling hospital spending. However, still in 2021, six regions exceeded the established ceiling. The AIFA reports indicate that some regions have found more difficulties than others adhering to national spending caps. In Campania, Calabria, Puglia, Abruzzo, and Lombardia the percent pharmaceutical spending relative to the total healthcare spending has been more elevated than in the other Italian regions (20, 21). Here, biosimilars availability could be instrumental to reduce spending associated to the purchase of high-cost medicines (e.g., biologic immunomodulators). However, uptake levels for TNF-alpha inhibitor biosimilars in Calabria and Abruzzo have remained low with respect to other areas, notwithstanding lower costs per DDD for biosimilars than for the originator (13).

#### 3.1.1. Supply-side considerations

On-patent immunosuppressive/immunomodulatory phar- maceuticals (e.g., vedolizumab, ustekinumab, secukinumab, tocilizumab, and ixekinumab) have been top contributors to hospitals pharmaceutical spending. Fortunately, the market presence of competing and more affordable TNF-alpha inhibitor biosimilars presented an opportunity to procure immunosuppressors/immunomodulators at lower cost. However, in the past, not every RHA included biosimilars within public tenders, and lots restricted to originators were commonly used to ensure therapeutic continuity for established patients (22). The interviewed experts mentioned that this has limited the market competitiveness, potentially reducing the savings potential after biosimilars availability. In 2017, a new Budget Law was approved, and the adoption of biosimilars was encouraged while respecting regulations that ensure patients’ therapeutic continuity (23). In line with these regulations, the AIFA considers that an originator biologic and its biosimilars are interchangeable, but that decisions on treatment initiation and switching ultimately belong to the prescriber. In this context, it is acknowledged by the AIFA that the regional health administrations and the hospitals can establish policies aimed at steering biosimilars use, provided that these policies help sustain cost-containment objectives.

It is established in the new Budget Law that when more than three medicines with the same active principle are marketed, public purchasing procedures using framework agreements need to be carried out. In the context of these agreements, originator and biosimilar pharmaceuticals with the same indications, dosage and administration routes have competed based on price. The outcome of this competition is described in a ranking list, where the top three products are the winners. The regional contracting authorities are bound to close a purchasing agreement with all the three winners (24). Interviewees indicated that this was a step forward in the direction of promoting the use of BVB, compared to previously established tenders restricted to originators. However, it was highlighted that the rules to use the ranking list are unspecific, which has led to different regional interpretations and partly explains heterogeneity in biosimilars adoption (25). It is pertinent to mention here that our data reflect high intra-country variability in TNF-alpha inhibitor biosimilars uptake in Italy (up to an 87%), even after 6 years of these products being available in the market. In fact, in some regions (e.g., Molise, Calabria, and Abruzzo) TNF-alpha inhibitor biosimilars uptake has been consistently low over time. For instance, the uptake of etanercept biosimilars in Molise only amounted to 13.6%, while the uptake in regions such as Bolzano, Valle d’Aosta and Piemonte was above 75%.

Regarding the use of the agreements ranking lists, one aspect that has led to confusion is the unclear criteria for the division of supply quotas among the three tender winners. Another important aspect that has not been clarified is whether prescribers should prefer the first-ranked drug within the group of winners. The jurisprudence of the Regional Administrative Courts (e.g., T.A.R in Puglia, Sardegna, Marche) seems to indicate that there should be no preference between the three winners. Under these conditions, the originator might be the second- or third-ranked product and still be preferred by providers over the first-ranked product. In some regions this may not be considered a problem, as the three contract winners are supposed to incur similar costs. Conversely, regional administrations that opted for implementing biosimilar quotas and switching protocols have had a preference to support biosimilars adoption, even in populations of biologic-experienced patients.

Even though the described purchasing framework allows plurality of providers, it is possible that the originator product may not be included in the list of tender awardees. In this sense, it has been a general concern to ensure affordable originator’s supply for the remaining populations of biologic-experienced patients that are not eligible or do not consent to a switch. Most regional administrations have addressed this issue by requesting drugs not awarded a contract to be purchased at the unit price offered by the Marketing Authorization Holder in the tender (this price should not be higher than the auction base). This has allowed to minimize the establishment of contracts outside of public tenders, and to avoid the purchase of originator drugs at prices higher than anticipated by regional administrations. In response, originator manufacturers have issued appeals to fight this system and have indicated that originator products should be excluded from tenders and be purchased via direct negotiations with hospitals.

#### 3.1.2. Demand-side policies for biosimilars use

To the best of the authors’ knowledge, most Italian regions (*n* = 17) have implemented measures aimed at steering biosimilars use. Across Italy, these measures were adopted at different moments and with heterogeneous content. Generally, policies implemented before the 2017 Budget Law and the release of AIFA’s statement endorsing switching, focused on fostering biosimilars use in biologic-naïve populations. In this line, already in 2009, the Campania region opted for the selection of biosimilars as first line treatments for naïve patients (26). Regions such as Friuli-Venezia-Giulia and Veneto followed this initiative and established biosimilar prescription quotas for newly diagnosed patients. The regions Basilicata, Calabria, Puglia and Sicilia legislated as well on the importance of preferring biosimilars over originator products, provided that the biosimilars were the most economically advantageous alternatives (27, 28).

Despite the readily implementation of biosimilar policies targeting biologic-naïve populations, the cost-savings potential to be achieved for the total drug budget with this relatively small group of patients has been limited. To extend the savings opportunity offered by biosimilars to biologic-experienced populations, the regional health administrations started regulating switching practices. The need to regulate these practices preceded, by more than 8 years, AIFA’s statement discussing the adequacy of switching. In 2010, for instance, the Toscana region recognized the possibility to switch biologic-experienced patients. However, this practice required a report by the prescriber indicating the reasons for the switch. Interviewed experts identified the need to justify a switch from an originator product to its biosimilar, as a barrier to biosimilars adoption. The effect of this barrier has persisted over time, and the publication of AIFA’s statement endorsing switching practices (2018) has helped in overcoming this effect. Most regions now require prescribers to justify the choice of an originator if a patient is eligible for a switch to a more cost-effective biosimilar (e.g., Toscana and Piemonte). In Piemonte for example, current biosimilar adoption guidelines incorporate prescribing objectives for BVB higher than the 95% (28). Compliance with these guidelines has been reflected in the achievement of 99.5 and 98.2% market shares for infliximab and adalimumab biosimilars, respectively.

Interviewees considered that incorporating biosimilar prescription objectives for biologic-experienced patients in regional guidelines, and clearly stating regulators’ expectations on biosimilars use, has helped hospitals in defining cost rationalization strategies. Further, asking prescribers to justify their therapy choices has minimized the number of cases where a less-affordable biologic was selected. However, this measure has faced the opposition of diverse stakeholder groups (including the originator’s industry and prescribers), and it has been argued in court whether it harms freedom of prescription. So far, regional tribunals have broadly considered that a justification request does not necessarily damage prescription freedom. Still, to avoid conflict in this respect, some regional administrations have opted to be less strict than others when it comes to asking prescribers to justify their choices. This difference in approaches has been reflected as well in the heterogeneous TNF-alpha inhibitor biosimilars uptake.

### 3.2. The TNF-alpha inhibitors market in Portugal

The five regional health administrations in Portugal (ARS: Alentejo, Algarve, Centro, Lisboa e Vale do Tejo, Norte) have the competence to manage primary services and supervise hospital care. However, most interventions aimed at regulating the pharmaceutical market concern the central health administrations. The National Authority of Medicines and Health Products (Infarmed) controls medicines market access and is involved in pricing and reimbursement decisions. Reimbursement for biosimilar medicines can be granted if the price of the first biosimilar’s entrant does not exceed 80% of the originator’s price, and if subsequent biosimilar entrants’ price does not surpass 70% of the originator’s price. Once biosimilars are approved for reimbursement, the role of the Central Administration of the Health System (ACSS) setting incentive mechanisms to support biosimilars use is decisive (18, 29).

#### 3.2.1. Demand-side policies for biosimilars use

ACSS coordinates the financial resources of the Portuguese National Health Service (SNS). Based on an existing framework for health services contracting, ACSS agrees with the ARS on budget limits for each year. These are specified in Contract-Programs that are later adapted for hospitals according to specific needs (30, 31). Adherence to these contracts is monitored by ACSS, which has additionally set incentives to ensure hospitals’ compliance with national access, quality and efficiency objectives. Increasing the percent use of biosimilar products is one of these quality objectives. Via a benefit-sharing mechanism, NHS hospitals that achieve at least 20% uptake for new biosimilar entrants in a year’s time can keep 15–25% of the generated savings for reinvestment. Conversely, non-compliance with the 20% quota can be penalized. It has been discussed whether the 20% threshold should be higher, as achieving it does not necessarily require concurrence with the ambitious switching objectives set as recommendations by the National Pharmacy and Therapeutics Committee (CNFT).

The CNFT (Infarmed specialized technical committee) has evaluated, on a molecule-by-molecule basis, scientific evidence on the safety and efficacy of using TNF-alpha inhibitor biosimilars. The current position is that treatment should be started with BVB (most likely a biosimilar) in all new patients. In the case of infliximab, etanercept, and adalimumab, there is enough evidence to assume that switches from the originator to biosimilars will not entail loss of efficacy or increased risk of adverse reactions. Consequently, according to the CNFT, switches to biosimilars should be organized for all clinically stable patients that have been given the originator medicine for at least 6 months (12). Hospital pharmacists interviewed for this study are favorable to the existence of CNFT switching guidelines, but pointed out two aspects that have affected early biosimilars adoption. First, the CNFT position statement on switching was only published in 2018 for infliximab and etanercept (32), and in 2021 for adalimumab. This implied a considerable time lag between biosimilars availability and switching guidance, and a missed opportunity to optimize cost-savings generation soon after biosimilars availability. Second, CNFT switching guidelines are to be viewed as recommendations.

According to the current regulatory framework, the ultimate responsibility to select an originator or a biosimilar medicine belongs to the individual prescriber, and there is no legal obligation to switch patients to the most cost-effective alternative. In this line, automatic substitution by the hospital pharmacy is not permitted. So, in the event of refusal to switch, the hospital pharmacy shall continue to provide the product that the patient was using, even if this product has not been awarded a supply contract by the hospital (33). In practice, it can be that despite recommendations by CNFT and the hospital Pharmacy and Therapeutics Committee (CFT), specific clinical departments may not agree to initiate a switching protocol for their patients. According to the interviewed hospital pharmacists, resistance of clinical departments to switch patients receiving treatment with subcutaneous etanercept and adalimumab has been higher than for intravenous infliximab products. This is reflected in the observed gap between infliximab biosimilars uptake national average, and the national average for etanercept and adalimumab biosimilars (see Figure 2). From the regulator’s point of view, and according to interviewed hospital pharmacists, biosimilar policies implemented so far have been successful at fostering the use of biosimilars used in acute care. For instance, uptake of granulocyte colony stimulating factors primarily indicated for the prevention/management of chemotherapy-induced neutropenia (e.g., filgrastim) reached 100% across Portugal. These policies have also facilitated overcoming initial resistances to switching. However, most hospital pharmacists interviewed have raised concerns regarding how to handle switches for patients receiving treatment with self-administered subcutaneous biologics (e.g., etanercept and adalimumab), and regarding multiple biosimilar-to-biosimilar switches. Current CNFT guidance regarding multiple switches indicates that multiple brand changes could increase the risk of loss of efficacy, and that in turn, this could lead to the loss of the economic advantage that justified the switch in the first place. For this reason, it is recommended not to make repeated brand changes over time. It is the responsibility of the hospital to decide when it is possible to proceed to a multiple switch, and what is the limit number of switches that a patient can undergo. This piece of guidance has been found to be vague by some HCPs, that have concerns about ensuring traceability of multiple switches. Hence, the focus of some hospitals on implementing biosimilar adoption strategies that improve the conditions for traceability of biologics throughout the whole utilization circuit (e.g., CHUC). In addition to providing tools to facilitate biologics traceability, the biosimilars strategy adopted by CHUC (i.e., University Hospital in Coimbra) has relied on: (1) communicating about the importance of following CFNT switching guidance; (2) defining a clear plan to foster biosimilars adoption, with specific objectives and timelines; (3) having a policy that prioritizes patients with external prescriptions being treated according to hospital cost-effectiveness standards (34). Uptake levels above 85% reached for all TNF-alpha inhibitor biosimilars in CHUC (in contrast to national uptake averages, see Figure 2) have shown the success of combining these strategies.

#### 3.2.2. Access to care limitations may affect biosimilar uptake patterns in Portugal

In addition to establishing efficiency and quality of care indicators, the National Health Administration gives priority to addressing challenges to access care. Literature analyzing the Portuguese Health System’s performance indicates that long waiting times are often an important access barrier. This can partly be attributed to an uneven distribution of healthcare resources across the country, with the coastal areas of Lisbon and Porto, and the metropolitan areas in the regions Norte and Centro, having more healthcare facilities and workforce (35). It is precisely in the metropolitan areas of Porto (e.g., CHSJ, CHVNGE, and CHPUVC) and Coimbra (e.g., CHUC) where TNF-alpha inhibitor biosimilars uptake has been generally higher (15). Conversely, interior and southern areas have had an increased reliance on private heath provision, for example to access specialized physician practices such as rheumatology practices. Years after the establishment of the Rheumatology Hospital Referral Network (RRH), designed to optimize coverage of rheumatology services across the country, access to these services in the south was still lower than in the region Centro (36). Interviewees have expressed that the higher reliance on private practices in the south/interior areas of Portugal, in relation to other metropolitan areas, could partly explain heterogeneity in TNF-alpha inhibitor biosimilars uptake. This is because the requirements for the preferential prescription of BVB in private prescribing centers can be less strict than in NHS hospitals, and because patients initiating treatment in these centers will eventually get their prescriptions or receive care from NHS hospitals. Patients that were initiated on non-BVB via a private practice would then necessitate a switch to comply with the hospital’s cost-effective prescribing standards. It is possible that the required switch may not be carried out in practice, due to the opposition of the prescriber and/or the patient. Although the CFNT has stated that their recommendation to start all biologic-naïve patients on BVB applies to NHS hospitals and to external prescribing centers, there are limited mechanisms for NHS hospitals to influence prescribing behavior outside of their institution (12).

#### 3.2.3. Supply-side considerations

To centralize and rationalize medicines procurement within the SSN, SPMS (Shared-services of the Portuguese Ministry of Health) organizes national public framework agreements and tender procedures. Oncological and immunomodulator products have generally been procured by SPMS based on volume needs estimated by hospitals, and via framework agreements (37). The latest agreement for these drug groups has been effective since 2018 (with a maximum validity of 3 years) and has been updated in 2021. Within the TNF-alpha inhibitors drug class, infliximab, etanercept and adalimumab originators were selected for procurement, as well as the corresponding biosimilars: Flixabi^®^, Inflectra^®^, Remsima^®^, Zessly^®^ (infliximab); Benepali^®^, Erelzi^®^ (etanercept); Amgevita^®^, Hulio^®^, Hyrimoz^®^, Idacio^®^, Imraldi^®^ and Yuflyma^®^ (adalimumab). Based on this selection, each hospital organized further procedures with suppliers, to determine specific purchase conditions (38). These procedures have been generally characterized by only considering price criteria, and sometimes by not allowing multiple winners. Interviewees have expressed concerns about this tender design and point to long-term sustainability risks if an excessive pressure is put on prices for biosimilar medicines. However, the situation where hospitals cannot afford purchasing multiple products containing the same active molecule is acknowledged and understood by interviewees. In light of these challenges, interviewees considered that future efforts to optimize procurement/purchase processes for biologics should focus on: (1) avoiding mechanisms that lead to excessive price erosion for biosimilars, and (2) incorporating multiple award criteria. Looking to the future, concerns have been raised as well regarding the establishment of hospital tenders that consider short time frames (i.e., shorter than a year). These have generated the need to set-up parallel purchase procedures to keep meeting the product’s demand, and that have led to higher than anticipated acquisition costs for the SNS. In turn, this affects biosimilars capacity to generate cost-savings within the system.

### 3.3. The TNF-alpha inhibitors market in Spain

In Spain, the primary jurisdiction over the management of health services and budgets has been transferred from the central government to the 17 largely autonomous regional health administrations. However, health spending objectives are set centrally, and regions that comply with these objectives can receive additional financial support (39). Medicines marketing, pricing and reimbursement decisions are also made centrally. The Spanish Agency of Medicines and Medical Devices (AEMPS) controls the marketing approval of pharmaceuticals, and the Interministerial Pricing Commission (CIPM) oversees pricing decisions, based on negotiations with manufacturers. Prices for biosimilar medicines must be lower than originators’ prices (generally 20–30% lower) and are set using reference list prices as in Italy, Portugal, and France (18). Reimbursement decisions emitted by the General Directorate of Pharmacy and Health Products for biosimilars are based on negotiations at the list price level. However, net prices paid by hospitals for pharmaceuticals are normally lower than the prices set by the CIPM, due to direct hospital-manufacturer negotiations, and to competitive purchasing procedures that can be established at the national, regional and local/hospital level in Spain.

#### 3.3.1. Supply-side considerations

The characteristics of regional and hospital tenders for TNF-alpha inhibitors vary across the country’s territory. To organize biologics procurement for the whole regional network of NHS hospitals, it has been common for regional health administrations to establish open tendering procedures via framework agreements (see 9/2017 Law for reference) (40). In these procedures, competitors are first ratified according to certain base conditions, and then selected for supply contracts. Since the market entry of biosimilars in Spain (2009), it has been discussed whether tender lots are to be defined at the molecule level, and consequently whether biosimilars and the originator product are to be grouped together. These discussions have been brought to court by industry representatives, which has meant a barrier to the time-efficient constitution of purchasing procedures for biosimilars (41). Considering that tenders can be awarded to a single winner, it has been argued that allowing biosimilar medicines to compete in the same lot as the originator could affect therapeutic continuity for patients being treated with an originator biologic. In this sense, interviewed hospital pharmacists indicated that prescribers have expressed concerns about purchasing processes for biologics that may steer therapy switches. To address these concerns, it has been discussed whether different purchase contracts should be used for populations of biologic-naïve and biologic-experienced patients. These arguments are to be understood in the light of an existing regulation that prohibits the substitution of biologic medicines by pharmacists (29/2006 Law, SCO/2874/2007). Historically, there has been hesitancy on whether this regulation applies only to community pharmacies or also to hospital pharmacies. In 2018, the AEMPs included a clarification on its website indicating that this law is to be applied at the community-pharmacy level (42).

It is now generally accepted that although biologics are not to be substituted by community pharmacists, the members of Pharmacy and Therapeutics Committees within hospitals can jointly decide on the interchangeability of biologics and agree on switching protocols for biologic-experienced patients. Based on the capacity to exchange biologics at the hospital pharmacy level, it has been possible for originator and biosimilar TNF-alpha inhibitors procured for hospitals to be placed in the same tender lot, and it has not been required to create different lots for biologic-naïve and biologic-experienced patients (41). It is to be noted that in practice, even though protocols for switching are established at the hospital level, and prescribers receive information on patients eligible for a switch, this cannot be mandated without the prescriber’s consent. In this sense, the behavior of specific clinical departments/prescribers may not reflect the intentions defined by the hospital Pharmacy and Therapeutics Committee. It has been discussed whether prescribers that do not select BVB for established patients should justify their choice. However, unlike some Italian regions, there is not a clear rule that makes this a central requirement.

Despite the possibility to place originator and biosimilar medicines within the same tender lot, this has not always happened for the market segment that corresponds to biologic-experienced patients in some regions/hospitals. In contrast, the standard purchase procedure in other regions has been to allow TNF-alpha originator and biosimilar products to compete for the whole market and based on multiple award criteria. In these cases, it has been possible to select multiple winners (e.g., Madrid) or only one winner (e.g., Andalucía) (43, 44). In general, interviewees with expertise on procurement procedures considered that the constitution of lots at the molecule level has maximized biosimilars potential to generate market competition and has facilitated biosimilars early adoption. It has also been indicated that awarding single-winner tenders to biosimilar manufacturers can drive biosimilars adoption in a more swift and uniform way. However, the latter strategy has raised concerns among procurement experts in Spain, regarding the high pressure put on prices and supply. Interviewees have also pointed to practices that have limited the capacity of biosimilars to compete for a share of the market. These are for example, cases in which purchase contracts (1–2 years’ duration) for originators (e.g., Remicade) were signed right before biosimilars market availability. This has been a problem when the system has not been able to adapt to biosimilars market entry and has been blocked for a period of time.

Regarding the organization of mechanisms for the central procurement of biosimilars, this has been the competence of The National Institute of Healthcare Management (INGESA). Since February 2020, the INGESA has been preparing a framework agreement proposal for the procurement of off-patent biologics and biosimilars. The regions can voluntarily decide whether they adhere to this agreement and therefore, whether they would be bound to derive supply contracts with the providers selected as a result of this procedure. This would be the first national-level agreement concerning the procurement of TNF-alpha inhibitor biosimilars. It has been clarified by the interviewed experts that the INGESA proposal is based on an open procedure that allows the plurality of bidders, and for which the award order would in principle only be based on price. Tender lots are to be organized at the molecule level and all the authorized pharmaceutical presentations are to be included. It has been indicated that the price offer presented by competitors could vary along the duration of the agreement (2 years), and that according to these variations, the award order could be continuously updated. For the procurement of TNF-alpha inhibitors, it has been estimated that discounts in the range of 36 to 58% could be awarded.

In principle, 10 out of the 17 Spanish regions (i.e., Aragón, Asturias, Islas Baleares, Cantabria, Castilla y León, Extremadura, Galicia, La Rioja, Región de Murcia, and Comunidad Valenciana), as well as the two autonomous cities Ceuta and Melilla decided to adhere to the INGESA framework agreement conditions. However, following the publication of the draft documents specifying these conditions, pharmaceutical industry representatives raised concerns regarding the continuous nature of the auction process. It was argued that allowing the award order to be updated according to new price offers, could lead to a downward spiral on prices. In parallel, HCPs and patients’ communities expressed concerns about the possible implications of this agreement on prescription freedom. The continuous nature of the proposed auction could indeed steer multiple therapy switches (45, 46). However, it is not yet clear whether these will be the real implications. Based on the raised objections to this procedure, it has been discussed whether a new procedure should be drafted. After 2 years of discussions, and the involvement of the Central Administrative Court on Contracts (TACRC) it is not yet clear how to proceed.

#### 3.3.2. Demand-side policies for biosimilars use

In the absence of concrete objectives for biosimilars use set nationally, almost all regional health administrations have issued recommendations to initiate biologic-naïve patients on BVB. However, a limited number of regions has specified a prescription target for naïve patients, and even a more limited number has endorsed switching practices based on cost-effectiveness criteria. The region Cataluña exemplifies a common situation, where the endorsement of protocols initiating naïve patients on BVB has been more active than the endorsement of switching protocols. In this line, ambitious biosimilar prescription targets (80%) have been set for naïve patients at the regional level since 2018, while specific targets have not been communicated for populations of biologic-experienced patients (47). Therefore, the main responsibility to provide guidance on switching practices has remained at the level of Pharmacy and Therapeutics Committees within hospitals. Conversely, more active regional approaches have been taken In Asturias, La Rioja and Andalucía to endorse switches based on cost-effectiveness criteria. In Asturias, the Technical Committee for the Rational Use of Medicines (CURMP) has established prescription protocols in rheumatology, to be implemented in all NHS hospitals. Based on cost-effectiveness criteria, these protocols have placed TNF-alpha inhibitor biosimilars as first line treatments after failure of conventional disease-modifying antirheumatic drugs (48). Compliance with these protocols is reflected in the uptake of etanercept biosimilars (92.2% in 2021), that has been considerably higher than the national uptake average for etanercept (53.7%). It is to be noted that most treatment guidelines for TNF-alpha inhibitor medicines’ use in Spain do not explicitly place biosimilars in a preferential location with respect to equivalent but less-affordable products. This is despite potentially high differences in acquisition costs at the hospital level, as indicated by Sanz-Alonso et al. (49).

It has been agreed by interviewees that having a national-level benchmarking system (like the Italian and Portuguese system) may help support biosimilars adoption (15, 50, 51). It has been difficult for hospitals to coordinate at the regional and national level, and to have uniformity of criteria regarding the promotion of TNF-alpha inhibitor biosimilars use. Publicly available data from the Comunidad de Madrid health outcomes observatory reflect these difficulties, with some hospitals reporting biosimilar uptake values below the 30%, and others within the same region reporting values above 75%. It is to be noted that not every region has a system that allows an easy comparison of biosimilars uptake at the hospital level and molecule level. In fact, the need for such systems has been indicated as a priority by interviewees.

## 4. Discussion

There is a need to explore the diverse market dynamics generated following biosimilars availability in Europe. So far, this exploration has led to a better understanding of why the competitive potential of biosimilars has not been deployed in every country across the whole range of marketed off-patent biologics. Our research has supported the identification of country-specific factors affecting biosimilars use. This with the ultimate goal of informing strategies to leverage biosimilars competition and that adapt to specific markets and care environments. To the best of the authors’ knowledge, the current study is the first to have comparatively evaluated TNF-alpha inhibitor biosimilars uptake levels regionally for Italy, Portugal, and Spain; and to have identified general and country-specific factors affecting biosimilars use within hospital markets in Southern-Europe (see Table 3). The use of a mixed methods methodology combining the quantitative analysis of market data with qualitative insights from experts is an added value of this study.

In line with published literature, our analysis shows that the widespread adoption of TNF-alpha inhibitor biosimilars has been limited by the indication of these products to treat chronic conditions (e.g., rheumatoid arthritis, ankylosing spondylitis, Crohn’s disease, ulcerative colitis, etc.). In the analyzed Southern European hospital markets, there is a clear difference between the evolution of uptake patterns for biosimilars used in chronic care and in acute care: patterns for acute care were found to be more homogeneous. For instance, in Portugal, while filgrastim biosimilars uptake was 100% across the whole territory (15), etanercept biosimilars national average uptake was below 50% (see Figure 2). This suggests that barriers to biosimilars adoption need to be understood in the context of the care area and the involved medical specialties. This aspect has been extensively discussed in the literature (3–6, 52). The contrast between biosimilar uptake patterns for products used in acute care versus chronic care also suggests that the barrier to be overcome does no longer concern HCPs and patients receiving basic information on the use of biosimilar medicines, but having their doubts on interchangeability and best switching practices effectively addressed. Additionally, this research points to the limited reach of biosimilar policies implemented locally to support switching practices, if not supported by central guidance and policy frameworks. In spite of the need for biosimilar policies targeting populations of biologic-experienced patients, the focus of policies implemented so far has been on biologic-naïve populations. As explained in the results section, this is reflected in the lack of prescription targets set for biologic-experienced patients, or in the set-up of targets for these populations that do not reach the 50%. It has also been reflected in the late release of switching guidance with respect to other European countries, and in the sometimes unspecific nature of this guidance. This has delayed the adoption of biosimilars at the hospital level in Portugal, Spain and in certain Italian regions, in cases where the regional health administrations have not had a pro-active approach toward the regulation of switching practices.

The aspects described above for Southern European countries contrast with the reality of other European countries. In Norway, the National Medicines Agency has clearly stated that “switching between reference products and biosimilars during ongoing treatment is safe” (no distinction in made between active molecules and formulations), and that “switching is necessary to achieve competition between equally efficient drugs.” A similar position has been supported by the Medicines Agency in Denmark. In these countries, where single-winner tenders for hospital-use biologics have been organized nationally, the switch between biologics with the same active substance has been automatic and has been mandated based on cost-effectiveness criteria (53, 54). In the context of single-winner tender procedures, biosimilar-to-biosimilar switches have been possible in these countries over time. The Norwegian Medicines Agency has referred to this situation, indicating in a position statement that switching between biologics of the same active principle is safe, and that this includes switching from a biosimilar to another biosimilar based on the same active principle. It is, however, clearly stated that all patients treated with a biological drug must receive the necessary follow-up. To ensure traceability, adverse reactions to biologicals should be reported specifying the drug name, the active principle and the batch number of the product (8, 55). In countries such as the UK and Ireland, a complete switch to the “best-value” alternative has not been sought. However, high quotas (80–90%) for BVB have been set-up for both, biologic-naïve and biologic-experienced patients (56, 57).

Our research has highlighted the variability in the implementation of switching and other biosimilar polices across Southern Europe and within jurisdictions. This variability can be explained in the context of policies being designed and implemented by the EU Member States and by regional governments within these States. Interviewed experts have pointed to the lack of guidance from the regulator’s side on interchangeability and best-switching practices as a barrier to biosimilars uptake. Overcoming this barrier may be less challenging now that the European Medicines Agency has emitted an official position statement discussing interchangeability considerations. Within this guidance document it is clearly stated “once a biosimilar is approved in the EU it is interchangeable, which means the biosimilar can be used instead of its reference product (or *vice versa*) or one biosimilar can be replaced with another biosimilar of the same reference product”(58). Having published this document is expected to support harmonization across Member States and within countries, but the actual impact is still to be evaluated. So far, in the absence of national-level harmonized interchangeability and switching criteria, our research indicates that hospitals’ success in rationalizing biologics spending has strongly relied on: (1) effectively raising awareness among HCPs on benefits offered by biosimilars; (2) the support of opinion leaders among clinical departments and the prescribers’ community; (3) achieving sufficient participants for voluntary managed-switch programs; (4) establishing incentives that can align the priorities of prescribers/patients and managers/payers. Our research suggests that primarily relying on these strategies, without having the support of a common comprehensive policy framework, leads to heterogeneous outcomes in the adoption of biosimilars. It is to be noted as well that there are limitations when it comes to optimizing these strategies. The success of raising awareness among HCPs on biosimilar benefits depends on the capacity to effectively communicate about real price differences between originators and biosimilars. Also, due to regulatory constraints, there is limited flexibility to design and implement incentives for HCPs at the hospital level in Italy, Portugal and Spain. The possibility to apply benefit-sharing strategies for biologics has already been explored in certain Italian regions and in Portugal, and there is room to further optimize these strategies (59). In Spain, the Biosimilar Medicines Association (BioSim) is currently discussing with regional health administrations and hospitals, how to best implement benefit-sharing practices (60).

Despite the added value of this research, some limitations can be attributed as well. First, we did not conduct interviews for all the relevant stakeholder groups in all the Portuguese, Italian and Spanish regions. This was due to difficulties reaching sufficient participation for research interviews. However, we believe that based on the profile of the included interview participants, and their vast experience on the topic, we were able to extensively describe supply- and demand-side considerations affecting TNF-alpha inhibitors use. Also, interview findings have been complemented with an extensive literature search. Although we discuss a series of biosimilar uptake determinants identified for Italy, Portugal and Spain, we did not investigate barriers for biosimilars adoption pertaining to patients’ perceptions on biosimilars. It has been extensively discussed in the literature that patients’ perceptions on biosimilars can influence biosimilars uptake (61–64). Further, it is possible that these perceptions are influenced by commercial strategies from companies (65, 66).

Second, due to missing data, regional TNF-alpha inhibitor biosimilar market shares were analyzed for different time frames in Portugal and Spain (2016–2021), and in Italy (2019–2021). Third, this study describes in a summarized way biosimilar policies and practices that can lead to low and heterogeneous biosimilars uptake. However, this is not meant to be a comprehensive description of all biosimilar policies and practices in use in each region of study. We acknowledge the relevance of studying, in addition to how biosimilars uptake levels evolve, the evolution of costs per molecule and patients treated. At the time of the analysis, these data were not publicly available at the regional level in the three countries of study. Future studies would benefit from reporting on these aspects. Findings from this study could be used as a starting point to explore determinants of biosimilars use for other therapeutic classes in Southern Europe. It would be interesting to investigate whether and how identified factors affecting the uptake of hospital-use biosimilars apply to molecules primarily used in the retail setting, to other care areas, and in general, to other countries.

## 5. Conclusion

In Italy, Portugal, and Spain, hospital pharmacists and managers are primarily responsible for fostering the preferential prescription of best-value TNF-alpha inhibitor biologics. In fulfilling this responsibility, they have been affected by the absence of common policies to steer biosimilars adoption, and by limited benchmarking and coordination capabilities. Health authorities’ position regarding best switching practices, the desirability of non-medical switching and how to manage multiple switches remains vague. The lack of guidance from the regulators’ side has affected biosimilars adoption in clinical practice. This study highlights the need for national/regional policy frameworks supportive of measures already implemented locally to foster biosimilars use in Italy, Portugal and Spain. These frameworks should account for the particularities of off-patent biologic and biosimilar markets and should jointly address supply- and demand-side challenges.

## Data availability statement

The datasets presented in this article are not readily available because they contain information that could compromise interviewees’ privacy and consent. This is applicable to interview datasets. Other original contributions presented in the study are included in the article. Further inquiries can be directed to the corresponding author. Requests to access the datasets should be directed to TBL, teresa.barcina@kuleuven.be.

## Ethics statement

The interview guide and methodology of this study was approved by the Research Ethics Committee UZ/KU Leuven (s65745).

## Author contributions

TBL, AV, IH, and SS were involved in the study conceptualization. TBL was involved in data collection and analysis and wrote the first draft of the manuscript. This implied performing the quantitative analysis of biosimilars uptake data and conducting/analyzing the interviews with Italian, Portuguese and Spanish experts (TBL has over 3 years of experience conducting market data analyses for biologics and with the semi-structured interviews technique). All authors critically reviewed the manuscript and approved its submission for publication.
