# Supplementary material for: An exploration of biosimilar TNF-alpha inhibitors uptake determinants in hospital environments in Italy, Portugal, and Spain
Source: Front Med (Lausanne). 2023 Jan 10;9:1029040. doi: 10.3389/fmed.2022.1029040 (PMC9871571; doi:10.3389/fmed.2022.1029040)
Supplement: Supplementary file 1 [file Data_Sheet_1.pdf]

Interview Guide

An Exploration of Biosimilar TNF-alfa Inhibitors Uptake Determinants in Hospital Environments in Italy, Portugal and Spain

Introduction – The introductory section of this interview guide has been summarized for inclusion in the Supplementary Materials section.

- **Present yourself**

Allow me to present myself. My name is [researcher's name] and I am [specify role as a researcher]. The research project that I am conducting on [study topic]. This study has been conceptualized by [indicate the name of the researchers]

- **Thank the interviewee for his/her participation in advance**

First of all, I would like to thank you for taking the time to participate in this interview. Your views, opinions and experiences are very important to our research.

- **Explain very shortly the purpose of the interview**

With this interview, we want to explore your experiences with the purchase/reimbursement/prescription/ use of biosimilars. This is to better understand market dynamics concerning TNF-alpha inhibitors in your country and to identify determinants of biosimilar uptake.

- **Put the interviewee at ease and explain the procedures for data processing.**
- **Indicate that the participation is voluntary and that it is possible to withdraw from the interview at any point, without having to give an explanation.**

We will start with some introductory questions and then, I will turn on the recorder and focus more on our specific study questions.

Questions

I. Introductory questions

1. Could you please start with introducing yourself?

1.a - What is your role/title within your institution? For how long have you held this title?

2. What has been your experience with the procurement, prescription and administration/use of biosimilars?

Let's start with the research questions now. Do I have permission to turn on the recorder?

II. General research questions

3. We learned from previous research that TNF-alpha inhibitors are generally prescribed and used in the hospital setting in (include the name of the country). Is this correct?

3.a - What biologic active principles are majoritarily administered in the outpatient setting? For these biologics, are they prescribed at the level of the hospital or at the level of specialized ambulatory centers?
3.b - What biologic active principles can be dispensed by community pharmacies?

Supply-side considerations

Pricing mechanisms for biosimilars

4. What kind of pricing system is in place for biosimilar medicines? (free pricing/price regulation)

4.a - What are the criteria for price setting?
4.b - How does the pricing system support/hinder biosimilar use?

About procurement mechanisms for biosimilars: *tendering vs. non-tendering and scope of tendering procedures.* *(Questions to be adapted to the country of study)*

When asking about the *characteristics of tenders*, we refer to: scope of tendering procedures, tendering award criteria, contract conditions and requirements for the reopening/renewal of tender contracts, etc.

5. What mechanisms are in place for the purchase of biologics to be used at the hospital level?

6. Are all the institutions in charge/qualified to purchase biologics aware of pricing negotiations happening at the central or regional level?

6.a - If not, how does this affect the purchase process?

7. Could you expand on the characteristics of national-level framework agreements for the purchase of biologics? Which molecules have been included? What have been the outcomes?

7.a - How have these framework agreements facilitated the use of biosimilars?

8. Could you expand on the characteristics of regional-level agreements for the purchase of biologics? Which molecules have been included? What have been the outcomes?

8.a - How have these agreements facilitated the use of biosimilars?

9. Regarding hospital-level tenders, what are their design characteristics?

9.a - How does the tendering design facilitate the use of biosimilars?

9.b - What is the proportion of biologics purchased outside of the standardized tendering mechanisms (via direct contracts/negotiations)?

10. In general, what would you say are the incentives established at the hospital level to procure medicines at low prices?

11. How is the physician's decision incorporated when tendering?

11.a - Can physicians maintain the therapeutic freedom to prescribe a product different than the tender product without having to justify their choice?

11.b- If the answer to 11.a is "no", ask the interviewee to provide more details about the process of justification.

12. How do these measures support/hinder biosimilar use?

About reimbursement procedures for biologics

13. How is reimbursement organized for biologic active principles for which biosimilars are available in the market?

14. Do reimbursement policies differentiate between best-value and less-affordable products having the same active principle?

14.a - Are there restrictions in the reimbursement of originator biologics once biosimilars enter the market?

<u>Demand-side considerations</u>

About biosimilar uptake levels

To provide a summary of the evolution of market shares for infliximab, etanercept and adalimumab (information to be adapted to the region represented by the interviewee).

15. To ask the interviewee to comment on this information and to confirm/ correct our data.

16. To ask the interviewee to comment on shifts in prescribing trends <u>from</u> TNF-alpha inhibitors exposed to competition <u>to</u> **(1)** on-patent TNF-inhibitors (Cimzia®, Simponi®); **(2)** other biologics with different mechanisms of action (e.g., vedolizumab); (3) other non-biologic therapeutic alternatives (e.g., JAK inhibitors).

17. To explain the range of variability in biosimilar market shares for the different regions. To ask the interviewee to explain regional differences in biosimilar market shares.

About initiatives established to support the use of biosimilars

18. Are there measures in place supporting the prescription of biosimilar medicines?

18.a – What are the measures in place? (e.g., prescribing guidelines, implementation of an electronic prescribing system, voluntary target agreements, compulsory prescription targets, budget caps and prescription audits, direct financial benefits, benefit-sharing strategies, direct financial penalizations, information materials/education).
18.b – Do these measures (specify according to answers to 18.a) differentiate between treatment-naïve and established patients?
18.c – How have these measures evolved over time?

19. Is there clear guidance for healthcare professionals regarding the criteria for interchangeability?

20. Is there clear guidance for healthcare professionals regarding switching procedures?

21. Which of the indicated supportive measures has been more effective?

21.a- If some measures were not effective, could you explain why?

III. Wrap up questions

22. What have been your main learnings regarding how to engage stakeholders in the use of biosimilars?

23. What steps should be/ are going to be taken in the future to support a sustainable market for off-patent biologics and biosimilars? Which measures should be implemented with priority?

IV. Closing remarks

These were all the questions that I had for you. I will turn off the recorder now. Thank you very much for participating.

24. Do you want to add anything else? or do you have any questions for us?
25. Do you think we overlooked some relevant questions/aspects?
26. Do you have suggestions on who else to contact in order to further expand our knowledge on this topic?

Conclusion

Thank you for your participation. If after this interview you would like to get in touch with us, please do not hesitate to send an e-mail.
